# The perks of a quality system in academia

**DOI:** 10.1016/j.nsa.2022.100001

**Published:** 2022-01-12

**Authors:** María Arroyo-Araujo, Martien J.H. Kas

**Affiliations:** Groningen Institute for Evolutionary Life Sciences, University of Groningen, the Netherlands

**Keywords:** Preclinical data quality, Quality system, Rigorous research practices

In recent years, the biomedical research community has reappraised the importance of rigorous research practices for delivering reliable data that can support the development of novel, safe and effective therapies (Bishop, 2019; Giles, 2006).

With the help of meta-analyses, it has become evident that the way preclinical studies are planned, conducted, and reported are often far from optimal and in some cases might even hamper the drug development pipeline. Published studies with poor validity (internal, external, and of construct) and vague reporting mislead researchers to build upon this flawed knowledge; this, in turn, slows down the development of new drugs as it inflates treatment efficacy expectations (Sena et al., 2014) and contributes to failed clinical trials. Furthermore, it has been suggested that lack of rigorous research practices and report transparency, together with our shortage of understanding of biological variables that explain between laboratory variation contribute to the low turnover of preclinical studies (Bespalov et al., 2021; Voelkl et al., 2018) which generates ethical concerns regarding the use of animals and public resources.

In an attempt to develop strategies for improving research practices in biomedical research, the European Quality In Preclinical Data (EQIPD) consortium was created through the European Union's Innovative Medicine Initiative (IMI). As part of the overall project, the EQIPD consortium has developed and released for public use a quality system (QS) meant to support the generation of robust and reliable data by promoting rigorous research practices (Bespalov et al., 2021).

The EQIPD-QS’ approach focuses on the design, conduct, analysis, and report of unbiased studies to deliver robust results and thus, contribute to the development of effective and safe therapeutic targets. Moreover, the system was designed to be applied in academic and industry research contexts.

Formal quality management is a solution commonly applied in industry. In academia, there are rather few examples of a quality management system applied to support basic and applied research. As we will illustrate below, the benefits of applying a user-friendly quality system such as the EQIPD-QS may be as obvious even in an academic environment as relevant.

This is especially true considering the somewhat underestimated large academic input into the drug discovery pipeline. This has become more evident over the years as indicated by the increase of FDA-approved drugs released by academia: from 24% in 1998–2007, up to 48% in 2011, and 55% in 2015 (Bryans et al., 2019; Patridge et al., 2015). Moreover, this increment has been achieved while pharma companies keep disappearing or decreasing their manpower, emphasizing the need for a quality system suitable for an academic context.

Thereby, the implementation of a QS such as EQIPD's could promote the formation of more conscientious researchers who can establish clear and efficient communication between labs, in and out of academia, while creating an integral scientific setting and improving the research culture in preclinical research. Therefore, our research group at the University of Groningen (the Netherlands) was one of the first to start implementing the EQIPD Quality System; this commentary aims to highlight important lessons learned.

A key factor for the successful implementation and maintenance of a quality system is a clear understanding of its purpose. Thus, we have formulated the reasons that motivated the implementation of the EQIPD- QS in our lab to which the reader might identify with (Table 1).

**Table 1:** Reasons to implement EQIPD-QS in a lab of the University of Groningen.

The burden of implementing a QS was minimized since The Netherlands has rather strict regulations regarding the use of animals for research so we felt that the QS would not imply much effort on top of our standard administration.
Academic research in Europe has become highly collaborative as diverse consortia illustrate. There is a need to facilitate the communication between partners to ensure the vision and goals of the project are achieved on time in full; collaborators must be able to rely on each other's performance, including scientific integrity matters.
One of the main tasks of a university is to train students and new researchers and to provide the best environment for this. If a QS promotes rigorous research practices to produce robust and meaningful data then, implementing such a QS would help our group to train pre and postgraduate students and better prepare them for a career as independent scientists.
While we used to think of research integrity as being related to falsification, fabrication, and plagiarism, current definitions of scientific misconduct adopted by The European Federation of Academies of Science and Humanities (ALLEA) and various universities have expanded and now include “good data practices” such as those related to data management and storage, improper research design, among others (Drenth, 2010).
There is a constantly increasing bureaucratic burden that is difficult to manage especially for the head of the lab who, in addition to management and supervision of students and staff, is expected to travel to meetings and collaborative partners, sits on various committees, etc. Having a systematic approach to oversee the lab's activities and progress is what also contributed to our decision.

The following part of this communication provides a brief overview of our experience implementing the EQIPD- QS, followed by the lessons learned.

In the case of our institute, as good research practices were already in place, the QS mainly served as a guide to assemble puzzle pieces. The documents that were needed to implement the QS *core requirements,* listed in Table 2, were divided into two main categories related to university general regulations or local lab guidelines.

**Table 2:** The 18 QS *core requirements* and approximate time spent in each of them.

Core Requirement	Time taken to complete				Described in a QS stand-alone document
	No time (the item was covered by an already existing document)	Minutes (around 30 ​min)	Hours (4–5 ​h)	Days (3–4 working days)	
1. Process owner must be identified for the Quality System		X			
2. Communication process must be in place	X	X			
3. The research unit must have defined quality objectives			X		
4. All activities must comply with relevant legislation and policies	X				
5. The research unit must have a procedure to act upon concerns of potential misconduct		X			
6. Generation, handling, and changes to data records must be documented				X	
7. Data storage must be secured at least for as long as required by legal, contractual, or other obligations or business needs	X				
8. Reported research outcomes must be traceable to experimental data	X				
9. Reported data must disclose all repetitions of the test regardless of the outcome	X				
10. Investigator must declare in advance whether a study is intended to inform a formal knowledge claim		X			
11. All personnel involved in research must have adequate training and competence to perform assigned tasks		X			
12. Protocols for experimental methods must be available	X				
13. Adequate handling and storage of samples and materials must be ensured	X				
14. Research equipment and tools must be suitable for the intended use and ensure data integrity	X				
15. Risk assessment must be performed to identify factors affecting the generation, processing, and reporting of research data		X			
16. Critical incidents and errors during study conduct must be analyzed and appropriately managed		X			
17. An approach must be in place to monitor the performance of the EQIPD Quality System, and address identified issues		X			
18. Resources for sustaining the EQIPD Quality System must be available		X			

The university regulations comprise records related to general topics relevant for preclinical studies such as the research code of conduct, the access and use of a data archiving system, the institute licenses for the use of animals and certain drug compounds, among others. In general, the content of these regulations hardly ever changes, and therefore these documents only had to be compiled once the system was adopted. In addition, since animal research regulations are so strict in The Netherlands this step only implied entering the already existing records to the QS *dossier* directory, so no further action was required.

As for the lab regulations, these documents and records address specific aspects of how research is planned and performed and kept up-to-date albeit with the changing staff. This includes the training of personnel, the compilation of standard operating procedures (SOP's), ethical approval protocols to perform specific experiments, etc. In some cases, this content had to be adapted to the templates provided by the QS, which made it clear and accessible, and also made any necessary later updates almost effortless.

Once all documents were identified, updated and/or created, they were sorted out following the system's guidance and recommendations until the completion of the implementation. The time taken to complete the *core requirements* of the QS is listed in Table 2.

When the implementation was finalized, a formal assessment meeting was carried out to evaluate the fitness of the QS implementation. Some documents were created during the implementation and together with lab reports they were shared in advance with the assessment team, while an overview of the expectations of the implementation was sent to us. There were two 2-h meetings in which a team of 3 assessors and two staff members from the university lab went through a checklist together. This checklist was focused on reviewing the fulfilment and fitness of the QS *core requirements* to our specific laboratory setting. In some instances, discussion and examples were exchanged between both parties to make sure the QS core requirements would hold valid for the different types of experiments and scenarios in our lab. At the end of the formal assessment, a series of recommendations to improve the fitness of the implementation were sent to us; these aimed to further promote and follow-up a positive research culture in our lab via day-to-day research practices overseen by the QS.

During the QS implementation, we came across different scenarios and we think the lessons learned from these are worth sharing.

Lesson # 1 - The devil is not so bad as he is painted

The time invested in the implementation of the quality system was limited since the lab was already operating at a high level. The implementation of EQIPD-QS does not necessarily result in more work where high standards are met while it strongly supports the generation of robust data.

Lesson # 2 – Quality System is a self-reflection tool

By following the step-by-step approach of the QS we identified unintended gaps in our training. In the case of our lab, MSc and Ph.D. students have a mandatory course addressing research integrity topics; however, there is no such course for Postdocs. This was easily solved by making the relevant university guidelines more easily accessible for all lab members to be properly informed.

Lesson # 3 – Do not hesitate to ask for help

As a university lab, we are required to archive all research data in the university repository. However, the administration of the repository entrusts the lab manager with approval to edit records in case of incomplete/mistaken back-ups; this goes against EQIPD-QS recommendations. Given that the university archive regulations go beyond any lab's reach, we contacted the department of Information Communication and Technology (ICT) and they easily modified the read/write permissions for our lab members. Solutions turned out to be much easier than feared and anticipated, and we only needed to ask for help.

Lesson # 4 – Facilitation of the onboarding process

Onboarding new employments in a research team and institute usually requires time. Having the QS in place provides a step-by-step guided process with documentation that can be followed by the new employee without overlooking important local, national, and/or international procedures and regulations. Moreover, interaction among co-workers within the research team who follow the same steps facilitates the onboarding process further by having a feedback system already in place.

Personally, the implementation of EQIPD-QS made me [M.A] aware of the urgent need to change the research culture for preclinical studies.

The familiarity with concepts like randomization and blinding shadows their importance on the eyes of scientists that easily forget to put them in practice and/or report whether they were carried out. Without specific guidelines on part of journals, missing items like these may go unnoticed until after publication; by then, it is difficult to assess the validity of the study and the worth of the resources invested. Likewise, published data with a high risk of bias tend to have a lower weight in meta-analysis studies, further limiting the contribution of the study.

In summary, implementing a QS such as EQIPD's in academia can promote the development of habits that boost the quality of executing and reporting preclinical research. We hope this empirical report will encourage fellow researchers to change the generally accepted way studies are usually conducted and reported so more meaningful results can be achieved in preclinical sciences.

## Funding

This project has received funding from the 10.13039/501100010767Innovative Medicines Initiative 2 Joint Undertaking under grant agreement No 777364. This Joint Undertaking receives support from the European Union's 10.13039/501100007601Horizon 2020 research and innovation programme and 10.13039/100013322EFPIA.

## Declaration of interest

None.
